# Short-term adverse effects of COVID-19 vaccines after the first, second, and booster doses: a cross-sectional survey from Punjab, Pakistan, and the implications

**DOI:** 10.1590/0037-8682-0044-2023

**Published:** 2023-06-02

**Authors:** Zia Ul Mustafa, Fareeha Maqbool, Mahnoor Wahid, Muhammad Salman, Shahzaib Haroon, Yusra Habib Khan, Tauqeer Hussain Mallhi, Brian Godman

**Affiliations:** 1 Discipline of Clinical Pharmacy, School of Pharmaceutical Sciences, Universiti Sains Malaysia, Penang, Malaysia.; 2 Department of Pharmacy Services, District Headquarters Hospital, Pakpattan, Pakistan.; 3 Department of Medicine, Faisalabad Medical University, Faisalabad, Pakistan.; 4 Department of Medicine, Nishtar Medical University, Multan, Pakistan.; 5 Institute of Pharmacy, Faculty of Pharmaceutical and Allied Health Sciences, Lahore College for Women University, Lahore, Pakistan.; 6 Department of Medicine, District Headquarters Hospital, Pakpattan, Pakistan.; 7 Department of Clinical Pharmacy, College of Pharmacy, Jouf University, Sakaka, Saudi Arabia.; 8 Department of Public Health Pharmacy and Management, School of Pharmacy, Sefako Makgatho Health Sciences University, Ga-Rankuwa, South Africa.; 9 Centre of Medical and Bio-Allied Health Sciences Research, Ajman University, Ajman, United Arab Emirates.; 10 Strathclyde Institute of Pharmacy and Biomedical Science, University of Strathclyde, Glasgow, UK.

**Keywords:** Adverse effects, COVID-19, Pharmacovigilance, Vaccines, Pakistan

## Abstract

**Background:**

Safety and efficacy concerns regarding coronavirus disease 2019 (COVID-19) vaccines are common among the public and have a negative impact on their uptake. We aimed to report the adverse effects currently associated with the vaccine in Pakistan to build confidence among the population for its adoption.

**Methods:**

A cross-sectional study was conducted in five districts of the Punjab province of Pakistan between January and March 2022. The participants were recruited using convenience sampling. All data were analyzed using SPSS 22.

**Results:**

We recruited 1622 people with the majority aged between 25-45 years. Of these, 51% were female, including 27 pregnant women and 42 lactating mothers. Most participants had received the Sinopharm (62.6%) or Sinovac (17.8%) vaccines. The incidences of at least one side effect after the first (N = 1622), second (N = 1484), and booster doses (N = 219) of the COVID-19 vaccine were 16.5%, 20.1%, and 32%, respectively. Inflammation/erythema at the injection site, pain at the injection site, fever, and bone/muscle pain were common side effects of vaccination. No significant differences were observed in the adverse effect scores between all demographic variables except for pregnancy (P = 0.012) after the initial dose. No significant association was observed between any variable and the side effect scores of the second and booster doses of the vaccine.

**Conclusions:**

Our study showed a 16-32% prevalence of self-reported side effects after the first, second, and booster COVID-19 vaccinations. Most adverse effects were mild and transient, indicating the safety of different COVID-19 vaccines.

## INTRODUCTION

Since World War II, the coronavirus pandemic has arguably been humanity’s greatest challenge. It has killed millions of people and severely impacted the economies of countries worldwide^1^ ^,^ ^2^. Pakistan, a low-middle-income country, has been significantly affected by the coronavirus disease 2019 (COVID-19)^3^ ^,^ ^4^. A vaccination campaign was initiated by the government on February 2, 2021 to curb the spread of COVID-19 in Pakistan^5^. The first phase targeted frontline health care workers (HCWs)^5^. Afterward, the government in Pakistan started mass vaccination campaigns by establishing multiple vaccine centers in every district of the country in hospitals and public places. It introduced a field force to help optimize the vaccination program. Subsequently, individuals above 60 years of age were vaccinated for free to increase the vaccination rates^6^. Vaccination efforts were then focused on the remaining population. Currently, all individuals aged >12 years are able to receive COVID-19 vaccines^7^. A variety of such vaccines are now available at vaccination centers in Pakistan. These include mRNA vaccines: Moderna and Pfizer-BioNTech; inactivated vaccines: Sinopharm and Sinovac; and non-replicating viral vector-based vaccines: AstraZeneca, CanSino/PakVac, and Sputnik^7^. 

Moreover, booster doses of COVID-19 vaccines are also available for free to immunocompromised individuals, HCWs, and the public^8^. This is because most of the population cannot afford the vaccine. The safety and efficacy of all COVID-19 vaccines mentioned above have been established in clinical trials^9^. With the considerable efforts of the Government of Pakistan, 71.62% of the country’s population has been fully vaccinated, and 75.68% of the population has received at least one dose as of November 7, 2022^10^. 

The efficacy of the COVID-19 vaccine and its contribution to herd immunity are well-established in the literature^11^ ^-^ ^14^. This is important in countries like Pakistan, which are under extreme pressure because of the economic consequences of public health measures, including lockdown measures, implemented to reduce the spread of COVID-19 and its consequences on morbidity and mortality in the absence of effective vaccines^15^ ^-^ ^17^. 

Vaccine hesitancy is common in Pakistan. It may impede mass immunization against COVID-19 despite the government’s initiation of an aggressive and comprehensive vaccination campaign with the establishment of vaccination centers in every town/locality^18^ ^-^ ^23^. Several factors contribute to vaccine hesitance, including conspiracy theories and mistrust of health authorities regarding the safety of vaccines^20^ ^,^ ^21^ ^,^ ^24^. Widespread speculation regarding serious adverse events following immunization^20^ ^,^ ^25^ and false news circulating on social media further undermine the COVID-19 vaccination campaign^22^ ^,^ ^26^. 

Successful vaccination campaigns require fundamental queries related to vaccine safety and efficacy to be fully addressed to increase the willingness of the population to receive COVID-19 vaccines^22^ ^,^ ^27^. In many parts of the world, the adverse effects of COVID-19 vaccines have been reported alongside reports of their efficacy in preventing severe illness, morbidity, and mortality^9^ ^,^ ^28^ ^-^ ^31^. However, only a few studies on the possible adverse effects of COVID-19 vaccines have been reported in Pakistan^32^ ^,^ ^33^. Consequently, we conducted this study to evaluate possible adverse events after the first, second, and booster doses of COVID-19 vaccines in Pakistan. This is important because early studies involving fewer patients typically do not sufficiently report adverse events. Therefore, coupled with concerns among the population regarding the speed of developing vaccines against COVID-19, post-vaccination monitoring of side effects is important to address any negative perceptions regarding COVID-19 vaccines, including misinformation. Such information is also important for health authorities and public health policymakers as they seek to improve acceptance rates because of the efficacy of the vaccines.

## MATERIALS AND METHODS

### Study design, participants, and setting

A cross-sectional study design was implemented to collect information on the adverse effects of COVID-19 vaccines. Data were gathered from the general population of Punjab between January and March 2022, and participants were recruited through convenience sampling in public locations. 

### Inclusion and exclusion criteria

Participants had to be >12 years, have received at least one dose of any COVID-19 vaccine in Pakistan, and be willing to participate in the survey. We excluded those who had not received any COVID-19 vaccine or refused to participate in the study.

### Study instrument

The instrument used in this study was adopted from a previous study conducted in Jordan^34^ with slight modifications after receiving appropriate permission from the corresponding author and reviewing the literature^25^ ^,^ ^28^ ^-^ ^33^. The leading investigators (ZUM and MS) prepared the initial draft of the study instrument. This draft was evaluated by a team of experts from the fields of medicine and pharmacy to determine the understanding and clarity of the content of the study tool. The data collection tool was revised based on the recommendations of the expert panel. The final draft of the study instrument comprised the following four sections: 

**Section I** contained seven questions about patient demographics, including sex, marital status, age, level of education, presence of any chronic disease, allergy to any food or medicine, and current status of influenza vaccination. 

**Section II** discussed the current status and types of COVID-19 vaccines administered. 

**Section III** comprised a list of possible adverse effects of COVID-19 vaccines, and participants were requested to select those they have experienced following COVID-19 vaccination. Hospitalizations due to adverse effects and the time of onset were also recorded.

**Section IV** presented information on the management of adverse events. This included the duration of symptoms and medication administration for any side effects encountered, which were subsequently recorded.

### Data collection procedure

The data-collection team administered questionnaires at different public locations. The investigators invited the general public to participate in the study after explaining the study objectives. Members of the data collection team interviewed those willing to participate. Children’s data were collected from their parents/guardians, and personal information was not collected from participants.

### Ethical consideration

All participants provided written informed consent prior to their enrollment in the study. For children (aged <18 years), consent was obtained from their parents before data collection. The participant’s data were coded and stored in a password-protected file accessible only to the researchers. The Human Research Ethics Committee of the Department of Pharmacy Practice, University of Lahore, approved this study (REC/DPP/FOP/UOL/68). 

### Data analysis

The SPSS version 22.0 for Windows was used for analyzing the data in this study. Categorical variables are presented as numbers and percentages, whereas continuous variables are presented as medians (interquartile ranges) and/or means (standard deviations). Adverse effect scores were calculated by adding the number of adverse effects reported by the study participants. The scores were subsequently compared between the demographic variables using the Mann-Whitney and Kruskal-Wallis tests^35^, where applicable. The aforementioned non-parametric tests ranked all values from low to high and compared the mean ranks. Statistical significance was set at P < 0.05.

## RESULTS

### Characteristics of the sample population

The investigators approached 1877 participants, of whom 1622 individuals were included in the study (response rate: 86.4%). Table 1 shows the demographic details of the sample population. Most of the study participants were aged 25-45 years, followed by 46-79 years (27.3%). Of these, 51% (N = 824) participants were females, including 27 pregnant females and 42 lactating mothers. Most of the study participants (33.2%) had a primary level of education, followed by 27.3% with a secondary education. Most patients (92%) had no underlying diseases or illnesses. Among those with comorbidities (N = 125), diabetes mellitus, arterial hypertension, and heart disease were the top three. None of the study participants reported being vaccinated against influenza. As shown in Table 1, most participants were immunized against COVID-19 with Sinopharm (62.6%) and Sinovac (17.8%). In the study population, 7% individuals did not complete the COVID-19 vaccination course. Furthermore, of the 1515 individuals who completed immunization, 219 received a booster dose of the COVID-19 vaccine (187 homologous and 32 heterologous booster shots).

**TABLE 1:** Characteristics of the sample population.

Variable	N (%)
**Age (years)**	
< 18	95 (5.9)
18-24	307 (18.9)
25-45	886 (54.6)
46-79	325 (20.0)
≥ 80	9 (0.6)
**Sex**	
Male	798 (49.2)
Female	824 (50.8)
**Education**	
Illiterate	163 (10.0)
Religious education only	260 (16.0)
Primary	538 (33.2)
Secondary	442 (27.3)
Higher secondary/diploma	195 (12.0)
Tertiary	24 (1.5)
**Marital status**	
Single	426 (26.3)
Married	1196 (73.7)
**Chronic diseases**	
No	1497 (92.3)
Yes	125 (7.7)
**Self-reported COVID-19 status**	
No, I have never been infected with COVID-19	1501 (92.5)
Yes, I got infected before receiving the vaccine	121 (7.5)
**COVID-19 vaccine**	
Sinopharm	1015 (62.6)
Sinovac	289 (17.8)
CanSino/PakVac	31 (1.9)
Pfizer-BioNTech	246 (15.2)
Moderna	7 (0.4)
Sputnik	3 (0.2)
AstraZeneca	31 (1.9)
**Dosing**	
Incomplete*	107 (6.6)
Complete**	1515 (93.4)
**Booster Dose (N = 1515)**	
Yes	219 (14.5)
No	1296 (85.5)
**Type of booster vaccine (N = 219)**	
Homologous	187 (85.4)
Heterologous	32 (14.6)
**Self-reported COVID-19 infection status post-immunization**	
Yes, I got infected after receiving the first dose of vaccine (N = 1622)	39 (2.4)
Yes, I got infected after receiving the second dose (N = 1484)	86 (5.7)
Yes, I got infected after receiving the booster dose (N = 219)	6 (2.7)

*Required a second dose of COVID-19 vaccine but did not take it; **Completed the recommended dosing schedule of vaccine (single dose vaccine = 31; 2-dose schedule vaccines = 1484)

### Incidence of side effects post-Covid-19 vaccination

**FIGURE 1:**
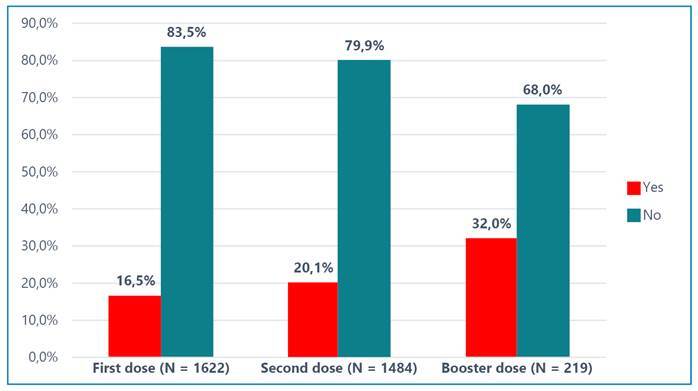
Frequency of side-effects after receiving the dose of COVID-19 vaccine.

**FIGURE 2:**
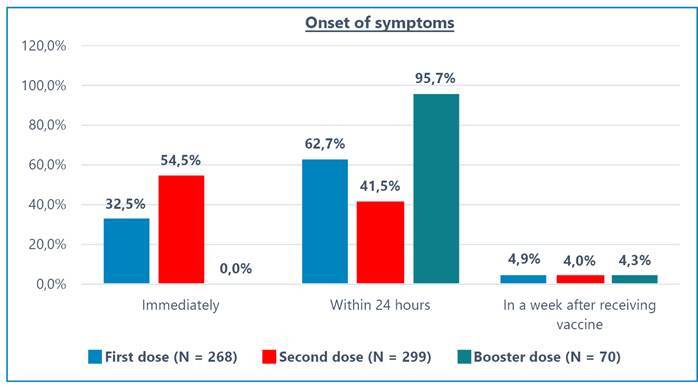
Onset of side effects after receiving COVID-19 vaccine.

Figure 1 shows that the incidences of at least one side effect after the first (N = 1622), second (N = 1484), and booster doses (N = 219) of the COVID-19 vaccine were 16.5%, 20.1%, and 32%, respectively. In most cases, the onset of side effects was within 24 h (Figure 2). Table 2 shows the complete side effect profiles of the COVID-19 vaccines. The respondents reported 28 different side effects after receiving the first vaccination and 16 after the second vaccination. Six side effects were observed after administration of the booster dose of the COVID-19 vaccine. 

**TABLE 2:** Adverse effects profile after receiving the COVID-19 vaccine.

	N (%)		
Adverse effects	First dose	Second dose	Booster dose
	(N = 1622)	(N = 1484)	(N = 219)
Inflammation and erythema at injection site	69 (4.3)	155 (10.4)	--
Pain at injection site	59 (3.6)	49 (3.3)	--
Fever	42 (2.6)	54 (3.6)	9 (4.1)
Bone and muscle pain	39 (2.4)	4 (0.3)	50 (22.8)
Fatigue	22 (1.4)	12 (0.8)	39 (17.8)
Headache	17 (1.0)	18 (1.2)	1 (0.5)
Palpitation	13 (0.8)	6 (0.4)	2 (0.9)
Sleep disturbances	10 (0.6)	1 (0.1)	--
Cough	9 (0.6)	5 (0.3)	1 (0.5)
Chills	7 (0.4)	4 (0.3)	--
Abdominal pain	7 (0.4)	1 (0.1)	--
Vomiting	6 (0.4)	--	--
Shortness of breath	5 (0.3)	--	--
Sore throat	4 (0.2)	3 (0.2)	--
Loss of appetite	4 (0.2)	--	--
Drowsiness	4 (0.2)	1 (0.1)	--
Runny nose	3 (0.2)	2 (0.1)	--
Nausea	2 (0.1)	--	--
Skin itching	2 (0.1)	--	--
Acne	2 (0.1)	--	--
Diarrhea	1 (0.1)	1 (0.1)	--
Constipation	1 (0.1)	--	--
Chest pain	1 (0.1)	--	--
Blurred vision	1 (0.1)	--	--
Excessive sweating	1 (0.1)	1 (0.1)	--
Anxiety	1 (0.1)	--	--
Depression	1 (0.1)	--	--
Decreased libido	1 (0.1)	--	--

The most common side effects after the first dose were inflammation or erythema at the injection site, pain at the injection site, fever, and bone and muscle pain. Similarly, inflammation or erythema at the injection site (10.4%), fever (3.6%), and pain at the injection site (3.3%) were the most frequently reported adverse effects of the second dose. However, patients who received a booster vaccine reported muscle and bone pain (22.8%), fatigue (17.8%), and fever (4.1%) within a day of receiving the booster dose (Table 3).

**TABLE 3:** List of patients with adverse effects requiring hospitalization.

Sr. No.	Gender	Age group	Vaccine type and dose	Reason for hospitalization
1	Female	25-45 years	SinoPharm	Muscle and joint pain + pain at injection site
			1^st^ dose	
2	Male	46-79 years	Sinopharm	Fever
			1^st^ dose	
3	Female	25-45 years	Sinopharm	Fever + chills + cough
			1^st^ dose	
4	Female	> 80 years	Sinopharm	Fever + fatigue/general tiredness
			1^st^ dose	
5	Female	46-79 years	Pfizer-BioNTech	Persistent headache
			1^st^ dose	
6	Male	18-24 years	Pfizer-BioNTech	High fever + chills +
			2^nd^ dose	cough + sore throat + headache
7	Male	25-45 years	Sinopharm	Fever + chills + fatigue + muscle and joint pain
			Booster shot	

The maximum number of side effects reported after the first, second, and booster vaccinations were four (mean = 0.21 ± 0.52), five (mean = 0.21 ± 0.45), and three (0.47 ± 0.78), respectively. No significant difference (P > 0.05) was observed in the side effect scores between all demographic variables except for pregnancy (P = 0.012), with pregnant women reporting more side effects with the COVID-19 vaccines after receiving the initial dose. We also found no significant difference in adverse effect scores between those who reported having COVID-19 before receiving the vaccine and those who did not (P = 0.457). No significant association was observed between any variable and the side-effect score following the second or booster doses of the vaccine.

### Need for hospitalization due to side effects associated with COVID-19 vaccination

Only five (0.3%) participants reported being hospitalized due to side effects after the first dose, while hospitalization due to side effects of the vaccine after the second and booster doses was 0.1% and 0.5%, respectively. Table 3 shows the details of the adverse effects of hospitalization. 

### Medications used to manage side effects of COVID-19 vaccines

The percentages of patients requiring medication to manage side effects after the first, second, and booster doses were 3.8% (N = 62), 2% (N = 30), and 15.1% (N = 33), respectively. Of the medicines prescribed to treat side effects, acetaminophen and non-steroidal anti-inflammatory drugs were the most common. 

## DISCUSSION

Our study revealed that most of the study population received the Sinopharm vaccine, followed by the Sinovac vaccine. This is because most of the imported COVID-19 vaccines were from China to initiate the COVID-19 vaccine program. In later stages, additional COVID-19 vaccines, including Pfizer-BioNTech, AstraZeneca, and Moderna vaccines, were imported from other countries under the Covax initiatives^33^. It was encouraging that most of the study population did not experience any adverse effects following the different COVID-19 vaccines. Inflammation and erythema at the injection site, followed by injection site pain, were the most common local adverse effects reported by those who received the primary COVID-19 vaccination series. The most frequent systemic adverse effects were fever and arthralgia/myalgia. The most frequent adverse effects of booster vaccinations were arthralgia/myalgia, fatigue, and fever. Our findings corroborate the results of clinical trials of various COVID-19 vaccines, most of which were administered to our study population. In the phase I/II trial of the Sinopharm vaccine, the most common adverse reactions were pain and fever, reported in a small number of vaccine recipients^36^. Furthermore, in phase III trials, most adverse events were mild to moderate in severity, with common adverse events such as pain at the injection site, headache, and fatigue^37^ ^,^ ^38^. In the combined safety profile of phase I and phase II trials of Sinovac, any adverse event within 28 days after injection occurred in 26% of participants in the 1.5 μg group, 29% in the 3 μg group, and 24% in the placebo group, without a significant difference (P > 0.05)^39^. Injection site pain was the most commonly reported local adverse event (1.5 μg group: 16%; 3 μg group: 16%; placebo group: 2%; P < 0.001), followed by inflammation at the injection site (1.5 μg group: 1%; 3 μg group: 3%; placebo group: 1%; P = 0.50). The most frequently reported systemic adverse effect was fever^39^. Sinovac’s phase III trial revealed that the frequency of any adverse event following immunization was 18.9%, compared with 16.9% in the placebo group (P = 0.018)^40^. In the clinical trial, the most frequent local adverse event associated with this vaccine was injection site pain, followed by erythema. However, the most common systemic adverse events were fatigue, headache, and myalgia^40^. In the randomized, placebo-controlled, observer-blinded trial of the BNT162b2 vaccine (Pfizer-BioNTech), the safety profile was characterized by transient mild-to-moderate injection-site pain, fatigue, and headache^41^. The incidence of severe adverse reactions was low and similar in vaccine and placebo recipients. 

The similarity of the results is encouraging and should provide reassurance to the population of Pakistan.

### Strengths and limitations

Our study had several limitations. We conducted this study in only five districts in Punjab. However, this province currently comprises over 50% of the population of Pakistan, and these districts were carefully chosen as representatives of Punjab. We recruited study participants through convenience sampling; consequently, there was a risk of bias inherent to this non-probability sampling technique. Additionally, the side effects were self-reported; therefore, participants’ information may have reporting and/or recall bias. Another important limitation was the high variability in the frequency of COVID-19 vaccines. It would have been ideal to compare the safety of the vaccines; however, this was not possible considering the different vaccines purchased by the Government of Pakistan.

 Furthermore, the main aim of this study was to reassure patients about the safety profile of the vaccines, given the current hesitancy rates and their rationale. We acknowledge that the number of people who received a booster vaccine was quite small in our study (N = 219), and it would be of great interest to conduct a large-scale study to compare the side effects associated with the first, second, and booster doses of COVID-19 vaccines in Pakistan. We will follow up on this topic. Despite these limitations, we believe that the findings of our study are encouraging and will help the general population build greater trust in the safety of COVID-19 vaccines in Pakistan to enhance future vaccination rates.

In conclusion, a low prevalence of adverse effects was generally observed among COVID-19 vaccine recipients in Pakistan. Most side effects (either local or systemic) were mild and transient. These findings indicate the short-term safety of COVID-19 vaccines for boosting purposes. This should help enhance future vaccination rates.
